# Impact of patient positioning on the efficacy and safety of doxycycline pleurodesis in malignant pleural effusion: a pilot randomized trial

**DOI:** 10.1186/s12890-026-04630-3

**Published:** 2026-08-31

**Authors:** Hieba Gamal Ezzelregal, Ahmed Mohamed Abdelsamad

**Affiliations:** Chest Department, Faculty of Medicine, Ain Shams University, Cairo, Egypt

**Keywords:** Pleurodesis, Patient Rotation, Malignant Pleural Effusion, Doxycycline, Thoracic Ultrasound

## Abstract

**Background:**

Chemical pleurodesis is a standard palliative intervention for recurrent malignant pleural effusion (MPE). Historically, patient rotation after sclerosant instillation was routine to ensure uniform drug distribution, but evidence supporting this practice remains conflicting. The study question was, does doxycycline pleurodesis without patient rotation demonstrate comparable efficacy and safety to the standard rotation protocol?

**Methods:**

This pilot randomized trial included 40 patients with recurrent MPE eligible for pleurodesis. Patients were randomized (1:1) to either a Rotation group (*n* = 20), which underwent a 2-h sequential rotation, or a Non-Rotation group (*n* = 20), which remained supine. The primary outcome was pleurodesis success at 1 month, assessed by chest ultrasonography. The secondary outcome was the Pleural Adherence Score (PAS) to objectively assess drug distribution and pain assessment.

**Results:**

The mean age was 56.5 years. Pleurodesis success rates were identical between the rotation and non-rotation groups 85% (17/20) vs. 85% (17/20); *P* > 0.99, though the confidence interval was wide (− 22.13% to 22.13%). Crucially, ultrasound-derived PAS scores at day 1 were high and comparable between groups (Mean 20.0 vs. 21.2; *P* = 0.33), confirming effective sclerosant distribution without rotation. There were no significant differences in pain scores, drainage duration, or complication rates between the two groups.

**Conclusion:**

Omitting patient rotation during doxycycline pleurodesis for MPE demonstrated comparable 1-month efficacy in this pilot study, though larger trials are needed to establish definitive equivalence. These findings support a simplified non-rotation protocol to reduce patient burden.

**Trial registration:**

The trial was retrospectively registered at ClinicalTrials.gov (Identifier: NCT07147270). ClinicalTrials.gov PRS: Home page (Modernized) published 08/29/2025.

**Supplementary Information:**

The online version contains supplementary material available at https://doi.org/10.1186/s12890-026-04630-3.

## Background

Malignant pleural effusion (MPE) represents a significant burden in advanced thoracic oncology, affecting approximately 15% of all cancer patients and profoundly impairing quality of life through dyspnea and pain [1, 2]. For patients with expandable lungs, chemical pleurodesis remains a cornerstone of palliative management, aiming to obliterate the pleural space and prevent fluid reaccumulation [3]. Among the various sclerosing agents, doxycycline has emerged as a widely accepted, cost-effective, and safe alternative to talc, particularly in resource-limited settings [4].

Despite the prevalence of this procedure, the optimal technique for sclerosant instillation remains a subject of historical debate. Traditionally, clinical protocols have mandated patient rotation—asking patients to move sequentially through supine, prone, and lateral positions—following agent administration [5]. This practice depends on the theoretical assumption that gravity-dependent rotation ensures uniform distribution of the sclerosant across the entire pleural surface, thereby maximizing pleural adhesions [6].

However, this mechanistic rationale has been challenged by radioisotope studies demonstrating that the sclerosant disperses diffusely throughout the pleural cavity regardless of patient positioning, likely driven by respiratory mechanics and capillary action rather than gravity [7]. Furthermore, the rotation protocol demands physical cooperation and is potentially painful for debilitated cancer patients, adding to the procedural burden without clear evidence of clinical benefit. While previous studies have examined this question using talc, data specifically addressing the necessity of rotation during doxycycline pleurodesis remain scarce [8].

This randomized controlled trial was conducted to compare the effectiveness and safety of a simplified non-rotation protocol to the standard rotation protocol in terms of doxycycline pleurodesis, by assessing pleurodesis success and procedural efficiency as potential benefits, and patient discomfort and procedure‑related adverse events as potential harms. We hypothesized that eliminating patient rotation would significantly decrease patient discomfort and enhance procedural efficiency.

## Methods

### Study design

This was a pilot randomized, parallel-group, comparative effectiveness trial conducted at the Department of Chest Diseases, at a tertiary university (Ain Shams University) hospitals, Cairo. Egypt, from May 2025 to December 2025. The trial was originally conceived to explore non-inferiority with a 20-percentage-point margin, though the sample size limits definitive conclusions. The study protocol was approved by the Institutional Ethics Committee (Approval No. FMASU R107/2025). All procedures performed in this study were in accordance with the ethical standards of the institutional research committee and with the Declaration of Helsinki. Written informed consent was obtained from all participants prior to enrollment. The trial was registered retrospectively after start of patient recruitment at clinicaltrials.gov with identifier: NCT07147270. The primary outcome, secondary outcomes, and statistical analysis plan were defined in the study protocol before enrolment, but public registration was delayed due to institutional administrative processes. The study finished all follow ups with the end of January 2026.

### Population

Patients with malignant pleural effusion participated in this trial as study subjects, but patients or members of the public were not involved in the design, conduct, reporting, or dissemination plans of the research.

Adult patients with histologically or cytologically confirmed malignant pleural effusion (MPE) were eligible for inclusion if they demonstrated an expandable lung on chest radiography following drainage and had an adequate performance status (ECOG ≤ 2). Patients were excluded if they had multiloculated pleural fluid, extensive pleural adhesions preventing lung re-expansion, active empyema, prior ipsilateral pleurodesis, or organ failure (liver, renal, or congestive heart failure). Additional exclusion criteria included severe coagulopathy, pregnancy, lactation, and prolonged use of corticosteroids or nonsteroidal anti-inflammatory drugs (NSAIDs). After being eligible, an accurate explanation of the procedure and the intervention, participants accepting to sign the written consent were enrolled.

### Sample size

A sample size of 26 patients (13 per arm) was calculated. The trial was designed to test whether the non‑rotation protocol was non‑inferior to the standard rotation protocol for pleurodesis success, using a non‑inferiority margin of 20 percentage points for the absolute difference in success rates (95% versus 85%), with a two‑sided alpha of 0.05 and 80% power. To account for potential dropouts and to increase precision, we planned to enroll 20 patients per group (total n = 40).

No interim analyses or formal stopping guidelines were planned for this trial.

### Randomization and blinding

Randomization was performed using a computer‑generated random allocation sequence created by an independent researcher not involved in patient care, with block randomization (fixed block size of 4) to ensure a 1:1 allocation ratio between groups. Allocation concealment was maintained using 44 opaque, sealed, sequentially numbered envelopes prepared by an independent researcher, who only had access to the sequence, but the clinicians who enrolled participants and assigned them to interventions did not have access to the sequence and could not predict the upcoming allocation. Envelopes were opened only after obtaining informed consent and confirming catheter patency for sclerosant instillation. While patients and operator were aware of the positioning protocol, the outcome assessor performing chest ultrasonography at 1 month was blinded to group assignment.

Blinding of the ultra-sonographer and data analyst was maintained by coding treatment groups as A and B in the dataset, without any description of the positioning protocol; apart from patient positioning during the 2‑hour dwell time, all other aspects of the pleurodesis procedure (catheter type, doxycycline dose, timing, and removal criteria) were identical between groups.

### Intervention

All procedures were performed in the ultrasound and thoracentesis unit of the Chest Department at Ain Shams University Hospitals by the authors who are experienced in pleural procedures (each having performed at least 50 ultrasound‑guided pleural drainages and pig tail insertions).

In both groups, pleurodesis was performed using a standardized protocol. All patients underwent ultrasound‑guided insertion of a 12–14 Fr pigtail catheter. When once‑daily drainage was < 150 mL, complete evacuation was confirmed by chest ultrasonography. To minimize discomfort, 20 mL of 2% lidocaine was instilled intrapleurally through the catheter 10 min before the sclerosant. Subsequently, 500 mg doxycycline diluted in 50 mL 0.9% saline was instilled; the catheter was flushed with 20 mL saline and clamped for 2 h.

Group A (rotation) underwent sequential repositioning every 30 min (supine → right lateral → prone → left lateral) during the 2‑hour clamp period. Group B (non‑rotation) remained supine throughout the 2‑hour clamp period. After the dwell time, the catheter was unclamped and allowed to drain, and was removed when output was < 100 mL/24 h.

Harms were pre‑specified as procedure‑related adverse events, including fever, empyema, respiratory failure, and death occurring during hospitalization or within 30 days after pleurodesis. Adverse events were assessed systematically by daily clinical evaluation and chart review during admission, telephone contact during the follow up month and by a scheduled hospital visit at 30 days. All adverse events were recorded in a standardized case report form and were summarized as the number and proportion of patients affected in each group.

### Outcomes

The prespecified primary outcome was pleurodesis success at 1 month, defined as a binary variable (success/failure). Success required both (1) absence of recurrent pleural effusion requiring repeat pleural intervention and (2) sonographic evidence of pleural adherence on thoracic ultrasound, quantified by the Pleural Adherence Score (PAS). PAS was also analyzed as a secondary outcome to characterize the magnitude of pleural adherence over time.

The PAS is a continuous score (9–27) obtained by thoracic ultrasound using a portable system (Mindray DP‑15; Mindray Bio‑Medical Electronics Co., Shenzhen, China) with a 3.5‑MHz convex probe and a validated 9‑zone scoring system. The hemithorax was divided into anterior, lateral, and posterior regions, each subdivided into upper, middle, and lower zones. In each zone, lung sliding was graded on a 3‑point scale (1 = evident sliding, 2 = equivocal/diminished sliding, 3 = absent sliding indicating adherence), and the PAS was calculated as the sum of all zone scores (range 9–27, higher scores indicating greater adherence). This differs from some previously published PAS systems that use 0–2 scoring per zone (0–18 total).

Pre‑specified secondary outcomes were: (1) total drainage volume (mL) from catheter insertion to removal, analyzed as a continuous variable; (2) duration of chest tube drainage (days) until removal, analyzed as a continuous variable; (3) pain intensity during the 2‑hour dwell time, measured once using a 0–10 Visual Analog Scale (VAS) and analyzed as a continuous variable; and (4) procedure‑related complications (fever, empyema, respiratory failure, or death) occurring during hospitalization or within 30 days, analyzed as proportions.

No important changes were made to the trial design, eligibility criteria, interventions, outcomes, or planned analyses after trial commencement.

The study protocol, including the full statistical analysis plan, has not been published but can be obtained from the corresponding author on request. Some about the study protocol available on Clinicaltrials.gov registry.

Data analysis was performed using IBM SPSS Statistics for Windows, Version 27.0 (IBM Corp., Armonk, NY). All randomized patients who received doxycycline pleurodesis and had all post‑procedure assessment were included in the primary analysis according to the modified intention‑to‑treat principle (mITT) or complete case analysis and were analyzed in the groups to which they were originally allocated. Binary outcomes (complications, success of pleurodesis) were compared between groups using the chi-square or Fisher’s exact test as appropriate, and effect sizes were expressed as risk differences with corresponding 95% confidence intervals was calculated to assess non‑inferiority against the pre‑specified margin of 20 percentage points. Continuous secondary outcomes (total drainage volume, duration of drainage, VAS pain score) were summarized as mean ± standard deviation or median (interquartile range) as appropriate and compared between groups using the independent‑samples t‑test or Mann–Whitney U test. Categorical harms (procedure‑related complications) were summarized as counts and percentages and compared using chi‑square or Fisher’s exact tests. Patients with incomplete key variables were excluded before analysis, and all included participants had complete data for the primary and secondary outcomes.

The study population were handled by complete‑case analysis; no imputation was performed. No subgroup or sensitivity analyses were pre‑specified, and none were conducted post hoc. A two‑sided p value < 0.05 was considered statistically significant.

## Results

**Fig. 1:**
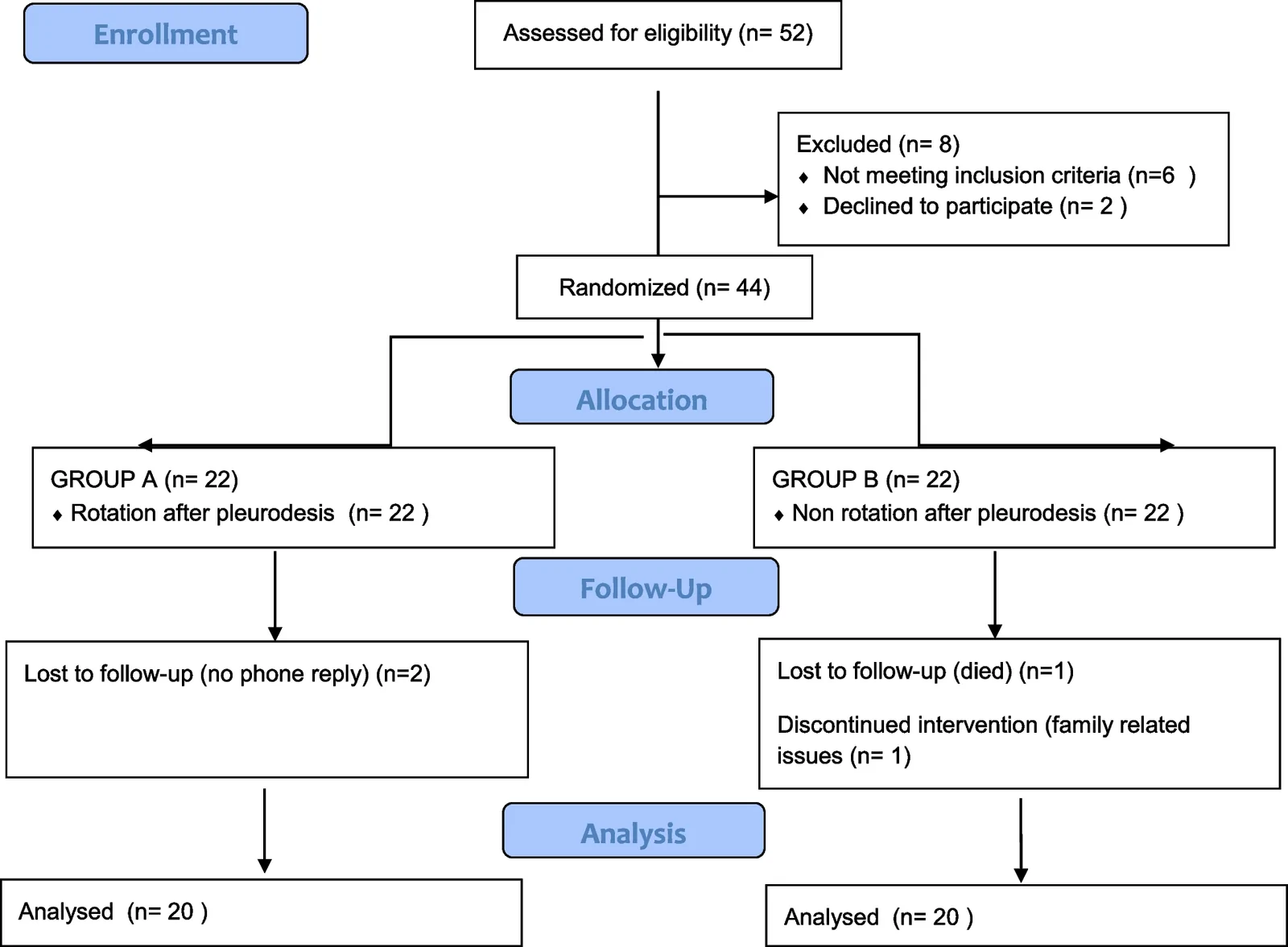
CONSORT flow diagram for participants’ recruitment

This pilot randomized conducted at the Department of Chest Diseases, at a tertiary university hospital, from May 2025 to December 2025. A total of 52 patients with malignant pleural effusion were assessed, of whom 44 were randomized, 4 were excluded and 40 were analyzed into two equal groups (*n* = 20 each) in the mITT analysis set with details shown in Fig. 1. The study finished all follow ups with the end of January 2026.

In both groups, pleurodesis was performed according to a standardized protocol. All patients underwent ultrasound‑guided insertion of a 12–14 Fr pigtail catheter. When daily drainage decreased to < 150 mL, complete evacuation of the pleural space was confirmed by chest ultrasonography. Doxycycline pleurodesis was then performed in an identical manner in both groups: the chest tube was clamped for 2 h after instillation, after which participants either followed the allocated rotation sequence or remained in the supine position, as per their randomized group. If any of the prespecified harms occurred, they were managed and treated as per guidelines as if the patient not included in the trial.

The mean age of the cohort was 56.5 years (SD 14.4), with a slight female predominance (57.5%). The most common histopathological diagnoses were metastatic adenocarcinoma (47.5%) and mesothelioma (45.0%). The pleura was the most frequent primary tumor site (45.0%), followed by breast (22.5%) and lung (15.0%). Baseline demographic and clinical characteristics appeared broadly similar between groups (Table 1).

**Table 1:** Baseline characteristics of study participants by procedure group

Characteristic	Rotation Group (*n* = 20)	No-Rotation Group (*n* = 20)	Test value	*P*-value^c^
Age (years), mean (SD)	60.6 (13.8)	52.4 (14.2)	1.866^a^	0.070
Sex, n (%)				
Female	12 (60.0)	11 (55.0)	0.102^b^	0.749
Male	8 (40.0)	9 (45.0)		
Histopathology, n (%)				
Mesothelioma	10 (50.0)	8 (40.0)	3.275^b^	0.513
Metastatic adenocarcinoma	9 (45.0)	10 (50.0)		
Metastatic melanoma	1 (5.0)	0 (0.0)		
Metastatic carcinoma	0 (0.0)	1 (5.0)		
Lymphoma	0 (0.0)	1 (5.0)		
Primary tumor site, n (%)				
Pleura	10 (50.0)	8 (40.0)	4.667^d^	0.458
Breast	4 (20.0)	5 (25.0)		
Lung	3 (15.0)	3 (15.0)		
Colon	2 (10.0)	0 (0.0)		
Kidney	0 (0.0)	2 (10.0)		
Others^d^	1 (5.0)	2 (10.0)		

*SD* standard deviation

^a^Independent t-test

^b^Chi-square test

^c^*P*-value > 0.05

^d^skin, larynx and blood

There were no significant differences in baseline pleural fluid characteristics (LDH, glucose, protein), ensuring comparable inflammatory states. Furthermore, the total volume of fluid drained, the amount of fluid drained before catheter removal, and the duration of chest tube drainage were statistically non-significant between the two groups (*P* value = 0.745, 0.309, 0.677, respectively), indicating that patient rotation did not facilitate fluid evacuation (Table 2).

**Table 2:** Pleural fluid characteristics and drainage outcomes by study group

Variable	Rotation Group (*n* = 20)	No rotation Group (*n* = 20)	Difference (mean ± SE (95% CI)	Test value	*p*-value^c^
LDH (U/L), median (IQR)	297.5 (228.5–354)	283 (216–306.5)	40.95 ± 35.47 (− 30.87 to 112.76)	−1.055^a^	0.291
Glucose (mg/dL), mean ± SD	104.6 ± 33.07	114.8 ± 34.34	− 10.20 ± 10.66 (− 31.78 to 11.38)	−0.957^a^	0.345
Protein (g/dL), mean ± SD	4.28 ± 0.91	4.36 ± 1.10	− 0.08 ± 0.32 (− 0.72 to 0.57)	−0.250^a^	0.804
Total fluid drained (mL), median (IQR)	3075 (2600–4150)	3225 (2550–4450)	− 110.0 ± 421.33 (− 962.94 to 742.94)	0.420^a^	0.677
Fluid drained before tube removal (mL), median (IQR)	0 (0–50)	50 (0–60)	− 8.50 ± 11.50 (− 31.78 to 14.78)	−0.325^a^	0.745
Full drainage time (days), mean ± SD	2.65 ± 0.75	2.55 ± 0.76	0.10 ± 0.24 (− 0.38 to 0.58)	−1.017^b^	0.309

*LDH* Lactate Dehydrogenase, *IQR* Interquartile range, *SD* standard deviation

^a^Independent t-test

^b^Mann-Whitney test

^c^*P*-value > 0.05: Non-significant

Thoracic ultrasound scores for pleural adherence were assessed at baseline, day 1, and 1-month follow-up (Table 3). Although the Non-Rotation group had slightly higher adherence scores at baseline (12.4 vs. 10.0, *P* = 0.009), this difference resolved after the intervention. At the primary endpoint of 1 month, both groups achieved comparable high levels of pleural adherence (21.9 vs. 21.4, *P* = 0.688). This confirmed that the omission of rotation not affected by the quality of lung expansion before pleurodesis, and also that no rotation did not compromise the 1-month adequate pleural apposition. To account for the baseline PAS imbalance, we analyzed changes in PAS over time. The increase in PAS from baseline to Day 1 was numerically greater in the rotation group than in the non‑rotation group (mean ΔPAS 10.0 ± 3.3 vs 8.8 ± 6.8), but this difference was not statistically significant (mean difference − 1.25; 95% CI − 4.7 to 2.2; *P* = 0.47). Similarly, the change in PAS from baseline to 1 month did not differ significantly between groups (11.9 ± 3.1 vs 9.0 ± 6.8; mean difference − 2.85; 95% CI − 6.3 to 0.6; *P* = 0.10). These ΔPAS analyses indicate that, despite higher baseline PAS in the non‑rotation group, the magnitude of pleural adherence gained after doxycycline pleurodesis was comparable between the two positioning protocols.

**Table 3:** Pleural adherence scores over procedure time

Time Point	Rotation Group (*n* = 20)	No-Rotation Group (*n* = 20)	Test value	*P*-value^b^
Baseline, mean (SD)	10.0 (0.7)	12.4 (3.8)	−2.763^a^	0.009
At Day 1, mean (SD)	20.0 (3.3)	21.2 (4.0)	−0.988^a^	0.329
At 1 Month, mean (SD)	21.9 (3.0)	21.4 (4.0)	0.404^a^	0.688
ΔPAS Baseline to Day 1, mean (SD)	10.0 (3.3)	8.8 (6.8)	− 0.739^a^	0.466
ΔPAS Baseline to 1 Month, mean (SD)	11.9 (3.1)	9.0 (6.8)	− 1.711^a^	0.099

*SD* standard deviation

^a^Independent t-test

^b^*P*-value > 0.05: Non-significant

We evaluated whether the Day 1 pleural adherence score predicted 1‑month pleurodesis success. Among 40 patients (34 successes, 6 failures), Day 1 PAS showed excellent discrimination, with (AUC 0.988; 95% CI 0.960–1.000; *P* < 0.001). Higher Day 1 PAS values were associated with greater likelihood of success, and cut‑offs between 17 and 19 provided high sensitivity and specificity; for example, a Day 1 PAS ≥ 17 yielded sensitivity 0.97 with specificity 0.83, whereas a cut‑off ≥ 18.5 provided sensitivity 0.91 with specificity 1.00. Given the small number of failures and the presence of ties, these findings should be interpreted cautiously but support the concept that early thoracic ultrasound adherence reflects subsequent pleurodesis outcome in our doxycycline protocol.”

In total, 40 patients were randomized equally to the rotation (*n* = 20) and non-rotation (*n* = 20) groups and all completed 1-month follow-up. Pleurodesis success and complication rates were identical in both groups: success 17/20 vs 17/20 with risk difference 0.0% [95% CI − 22.13% to 22.13%], (odds ratio 1.00 [95% CI 0.18–5.67]), *P* = 1.00. As regard complications 3/20 vs 3/20 with risk difference 0.0% [95% CI − 22.13% to 22.13%], (odds ratio 1.00 [95% CI 0.18–5.67]), *P* = 1.00. However, the 95% confidence interval for the primary outcome (pleurodesis success) crossed the prespecified non-inferiority margin of − 20 percentage points, so non-inferiority of the non-rotation strategy was not formally demonstrated.

The mean procedure-related pain score (VAS) was 6.1 (SD 2.0) in the Rotation group compared to 5.75 (SD 1.6) in the Non-Rotation group, mean difference − 0.35, 95% CI − 1.49 to 0.79; *p* = 0.54), indicating no statistically or clinically important difference in pain between the rotation and non‑rotation protocols. The median hospital length of stay was 3.0 days (IQR 3.0–4.0) and 4.0 days (IQR 3.0–4.0) for the Rotation and Non-Rotation groups, respectively (*P* = 0.122). (Table 4).

**Table 4:** Primary outcome, Secondary outcome and Complications

Outcome	Rotation Group (*n* = 20)	Non-Rotation Group (*n* = 20)	Effect measure (non rotation − rotation) (95% CI)	Test value	*P*-value^e^
Primary Efficacy Outcome, n (%)					
Pleurodesis success (1 Month)	17 (85.0%)	17 (85.0%)	0.0(− 22.13% to 22.13%)^d^	0.000^b^	> 0.99
Secondary and safety Outcomes					
VAS Pain Score, mean (SD)	6.1 (2.0)	5.75(1.55)	− 0.35 (− 1.49 to 0.79)	0.619^a^	0.540
LOS (days), median (IQR)	3.0 (3.0–4.0)	4.0 (3.0–4.0)	+ 1.35 (− 0.28 to 2.98)	−1.545^c^	0.122
Any Complication	3 (15.0%)	3 (15.0%)	0.0(− 22.13% to 22.13%)^d^	0.000^f^	> 0.99
Specific Complications Types^g^					
Pneumothorax	1/3 (33.3%)	2/3 (66.7%)		3.333^b^	0.343
Pneumomediastinum	1/3 (33.3%)	0 (0.0%)			
Hydropneumothorax	1/3 (33.3%)	0 (0.0%)			
Empyema	0 (0.0%)	1/3 (33.3%)			

*IQR* Interquartile Range, *VAS* Visual Analog Scale

^a^Independent t-test

^b^Chi-square test

^c^Mann-Whitney test

^d^Risk difference

^e^*P*-value > 0.05

^f^Fisher’s exact test

^g^specific complications represent the proportion among patients who experienced any complications

Regarding safety in Table 4, the overall complication rate was identical at 15.0% (3 patients) in each group (*P* = > 0.99). Specific complications in the Rotation group included pneumothorax (*n* = 1), hydropneumothorax (*n* = 1), and pneumomediastinum (*n* = 1). In the Non-Rotation group, complications included pneumothorax (*n* = 2) and empyema (*n* = 1). No mortality directly related to the procedure was recorded in either group. No additional subgroup or sensitivity analyses were performed beyond the primary and secondary outcome analyses reported above.

## Discussion

In this pilot randomized controlled trial, we found that omitting post-instillation patient rotation yielded comparable 1-month success rates to standard rotation (85% vs 85%)., with no meaningful differences in hospital length of stay, total drainage volume, or time to catheter removal. These findings indicate that the traditional practice of rotating patients after sclerosant instillation is not required to achieve effective pleurodesis.

Our results are concordant with accumulating evidence that patient rotation is an unnecessary step in chemical pleurodesis. The original rationale for rotation was to promote uniform distribution of sclerosant over the pleural surface; however, radiotracer studies have challenged this assumption. Dryzer et al. [5] used radio-labelled tetracycline and demonstrated rapid, near-instantaneous dispersion of the sclerosant throughout the pleural space irrespective of patient position. Mager et al. [6] similarly showed, using radio-labelled talc, that rotation did not affect clinical outcomes, with equivalent success rates in rotated and non-rotated patients (85% vs 85%), closely mirroring the 85% success rates observed in our trial. By confirming these findings in the context of doxycycline, our study supports the concept that physiologic pleural fluid dynamics and respiratory motion are sufficient for adequate sclerosant distribution without the need for manual repositioning [5].

A distinctive strength of our work is the incorporation of thoracic ultrasound to objectively quantify biological efficacy using the Pleural Adherence Score (PAS). Previous studies using a 0–18 PAS scale have shown that higher early adherence scores are associated with durable pleurodesis success [9, 10, 11] Because our scoring range (9–27) is not numerically identical to those systems, the specific thresholds reported in prior work cannot be directly transferred to our data. In the present trial, we therefore interpreted PAS as a continuous measure of adherence and focused on between‑group differences and changes over time rather than on any fixed cut-offs. Unlike earlier rotation trials that relied primarily on chest radiography, we used a direct ultra-sonographic marker of pleural symphysis. Although the non-rotation group had a slightly higher baseline PAS, both groups exhibited a marked and comparable increase in PAS by day 1 (mean 20.0 vs 21.2), with similarly high scores sustained at 1 month. Another scoring system has been previously validated, with early cessation of lung sliding shown to predict durable pleurodesis success in malignant pleural effusion [10, 12]. This sonographic trajectory provides robust physiological evidence that rotation does not enhance pleural inflammation or fibrosis beyond that achieved with standard doxycycline pleurodesis.

Consistent with previous studies suggesting that early ultrasound findings predict pleurodesis durability, our exploratory ROC analysis showed that Day 1 PAS was strongly associated with 1-month pleurodesis success with high discriminative performance (AUC 0.99), although this likely overestimates the true predictive accuracy and must be interpreted with caution given the small sample size.

Our findings also align with the recent randomized trial by Ekpe et al., [13] who evaluated rotation versus no rotation during tetracycline slurry pleurodesis in malignant pleural effusion. They reported no significant difference in pleurodesis success between rotated (92%) and supine (88.5%) patients, despite using tetracycline powder with relatively low water solubility. By demonstrating comparable results with doxycycline [14], which is now more widely used, and by adding objective ultra-sonographic confirmation of pleural adherence, our trial complements and extends these data. Taken together, these studies provide convergent clinical and physiological evidence that the routine use of patient rotation during chemical pleurodesis can be safely abandoned.

With respect to safety, omission of rotation did not increase procedure-related complications. The overall incidence of adverse events was low and similar between groups, and the single empyema case in the non-rotation arm resolved with antibiotics and prolonged drainage without surgical intervention. Rotation has been hypothesized to exacerbate pain due to repeated repositioning; however, mean VAS pain scores did not differ significantly between groups, suggesting that nociception is driven predominantly by the chemical pleuritis rather than by mechanical movement. Furthermore, rotation can be logistically challenging and uncomfortable, particularly in patients with advanced malignancy, dyspnoea, or frailty. The comparable median hospital stay of 3–4 days in both groups supports the conclusion that eliminating rotation neither delays discharge nor increases resource utilization.

This study has several strengths. The pilot randomized design reduces selection bias and supports causal inference regarding the lack of benefit from rotation. Use of thoracic ultrasound and a validated PAS represents a methodological advance over prior work, offering real-time, objective confirmation of pleural symphysis. In addition, we used a standardized protocol with a fixed doxycycline dose and uniform drainage criteria, limiting treatment heterogeneity.

While our point estimates were identical, the wide confidence interval (− 22.13% to 22.13%) reflects insufficient precision to definitively exclude clinically meaningful differences within our prespecified margin. This represents an exploratory finding requiring validation in larger trials. Several important limitations should be acknowledged. The most important, the sample size of 40 patients was insufficient to definitively establish non-inferiority within our prespecified margin of 20 percentage points. Recruiting adequate numbers in this specific patient population was challenging due to several factors: (1) patients with malignant pleural effusion had short survival, limiting the window for recruitment and follow-up; (2) our restrictive eligibility criteria required expandable lung, adequate performance status, and absence of multiloculated effusion. Single-center study conducted at tertiary academic hospitals all belong to Ain Shams University, which may limit generalizability to other settings with different patient populations, clinical expertise, or resource availability. The procedures were performed by experienced pulmonologists; results may differ in centers with less expertise in ultrasound-guided pleural procedures. Blinding of patients and treating clinicians to rotation versus non-rotation was not feasible, which may introduce performance bias, although outcome assessment was largely objective. Follow-up was restricted to 1 month; while this interval is standard for assessing early pleurodesis success, longer-term recurrence and need for re-intervention were not captured. Also the imbalance in base line PAS between groups which tried to be fixed by calculating delta PAS. Finally, procedural limitation is that trial registration on ClinicalTrials.gov was performed retrospectively in August 2025, after patient recruitment commenced in May 2025. This represents a deviation from ICMJE standards and best practices.

In conclusion, patient rotation after doxycycline instillation does not improve sclerosant distribution, pleural adherence, or clinical success in malignant pleural effusion. Thoracic ultrasound findings in this trial confirm that physiological respiratory motion and pleural fluid dynamics are sufficient to achieve effective pleurodesis without positional maneuvers. Given the absence of demonstrable benefit and the potential for added discomfort and logistical complexity, we advocate discontinuing the routine practice of patient rotation during chemical pleurodesis. Simplified, patient-centered protocols that maintain efficacy and safety while reducing procedural burden should be adopted as standard care in the management of malignant pleural effusions.

## Supplementary Information


Supplementary Material 1.
Supplementary Material 2.
Supplementary Material 3.
Supplementary Material 4.
Supplementary Material 5.
Supplementary Material 6.


## Data Availability

The datasets generated and analyzed during the current study are available in the Zenodo repository with DOI https://doi.org/10.5281/zenodo.18757942.

## Abbreviations

BMI: Body Mass Index
ECOG: Eastern Cooperative Oncology Group (Performance Status)
IQR: Interquartile Range
LDH: Lactate Dehydrogenase
MPE: Malignant Pleural Effusion
NSAIDs: Non-Steroidal Anti-Inflammatory Drugs
PAS: Pleural Adherence Score
SD: Standard Deviation
SPSS: Statistical Package for the Social Sciences
US: Ultrasound
VAS: Visual Analog Scale
