# Supplementary material for: Impact of patient positioning on the efficacy and safety of doxycycline pleurodesis in malignant pleural effusion: a pilot randomized trial
Source: BMC Pulm Med. 2026 Aug 31;26:384. doi: 10.1186/s12890-026-04630-3 (PMC13527988; doi:10.1186/s12890-026-04630-3)
Supplement: Supplementary file 1 [file 12890_2026_4630_MOESM2_ESM.pdf]

Expand All | Collapse
Home
Info and Help
Language/Options/Settings
Calculator
Counts
Std.Mort.Ratio
Proportion
Two by Two Table
Dose-Response
R by C Table
Matched Case Control
Screening
Person Time
1 Rate
Compare 2 Rates
Continuous Variables
Mean CI
Median/%ile CI
t test
ANOVA
Sample Size
Power
Random numbers
Searches
Google--Internet
PubMed--MEDLARS
Internet Links
Download OpenEpi
Development
Start
Enter
Results
Examples
Help

2 x 2 Table Statistics

Single Table Analysis

		Disease		
		complications	no complications	
	rotation	3	17	20
Exposure	no rotation	3	17	20
		6	34	40

Chi Square and Exact Measures of Association

Test	Value	p-value(1-tail)	p-value(2-tail)

Uncorrected chi square	0	0.5000	>0.9999999
Yates corrected chi square	0.1961	0.3290	0.6579
Mantel-Haenszel chi square	0	0.5000	>0.9999999
Fisher exact		0.6693	>0.9999999
Mid-P exact		0.5000	>0.9999999

At least one expected value (row total*column total/grand total) is < 5
Fisher or Mid-P exact tests are recommended rather than chi square.

Risk-Based* Estimates and 95% Confidence Intervals
(Not valid for Case-Control studies)

**Point Estimates**		**Confidence Limits**	
**Type**	**Value**	**Lower, Upper**	**Type**
Risk in Exposed	15%	4.394, 36.88	Taylor series
Risk in Unexposed	15%	4.394, 36.88	Taylor series
Overall Risk	15%	6.678, 29.45	Taylor series
Risk Ratio	1	0.2287, 4.372[1]	Taylor series
Risk Difference	0.0	-22.13, 22.13°	Taylor series
Etiologic fraction in pop.(EFp)	0.0	-73.77, 73.77	
Etiologic fraction in exposed(EFe)	0.0	-100, 77.13	
Prevented fraction in pop.(pfp)	0.0	-281.2, 42.45	
Prevented fraction in exposed(pfe)	0.0	-337.2, 77.13	

Odds-Based Estimates and Confidence Limits

**Point Estimates**		**Confidence Limits**	
**Type**	**Value**	**Lower, Upper**	**Type**
CMLE Odds Ratio*	1	0.1522, 6.572[1]	Mid-P Exact
		0.1167, 8.569[1]	Fisher Exact
Odds Ratio	1	0.1763, 5.673[1]	Taylor series
Etiologic fraction in pop.(EFp|OR)	0.0	-86.78, 86.78	
Etiologic fraction in exposed(EFe|OR)	0.0	-100, 82.37	
Prevented fraction in pop(PFpOR)	0.0	-656.6, 46.46	
Prevented fraction in exposed(PFeOR)	0.0	-467.3, 82.37	

*Conditional maximum likelihood estimate of Odds Ratio

(P)indicates a one-tail P-value for Protective or negative association; otherwise one-tailed exact P-values are for a positive association.
Martin,D; Austin,H (1991) An efficient program for computing conditional maximum likelihood estimates and exact confidence limits for a common odds ratio. Epidemiology 2, 359-362.
° [1] 95% confidence limits testing exclusion of 0 or 1, as indicated
P-values < 0.05 and confidence limits excluding null values (0,1, or [n]) are highlighted.
LookFirst items: Editor's choice of items to examine first.

Results from OpenEpi, Version 3, open source calculator--TwobyTwo
Print from the browser with ctrl-P
or select text to copy and paste to other programs.

It looks like there are no examples for this exercise.
